# Polyphenols and Eye Health: A Narrative Review of the Literature on the Therapeutic Effects for Ocular Diseases

**DOI:** 10.3390/nu18010069

**Published:** 2025-12-25

**Authors:** Angela D’Angelo, Giuseppe Giannaccare, Filippo Lixi, Mario Troisi, Ginevra Giovanna Adamo, Deokho Lee, Ilaria De Pascale, Alfonso Pellegrino, Livio Vitiello

**Affiliations:** 1 Department of Clinical Sciences and Community Health, Department of Excellence 2023–2027, University of Milan, 20122 Milan, Italy; angela.dangelo@unimi.it; 2 Department of Epidemiology & Biostatistics, Joe C. Wen School of Population & Public Health, Susan & Henry Samueli College of Health Sciences, University of California, Irvine, CA 92697, USA; 3 Eye Clinic, Department of Surgical Sciences, University of Cagliari, 09124 Cagliari, Italy; giuseppe.giannaccare@unica.it (G.G.); f.lixi0106@gmail.com (F.L.); 4 Eye Clinic, Department of Neurosciences, Reproductive and Odontostomatological Sciences, University of Naples Federico II, 80138 Naples, Italy; troisi165@gmail.com; 5 Ophthalmologic Unit, University Hospital of Salerno, 84131 Salerno, Italy; 6 Department of Translational Medicine, University of Ferrara, 44121 Ferrara, Italy; dmagvr@unife.it; 7 Sant’Anna University Hospital, 44124 Ferrara, Italy; 8 The Korean Institute of Nutrition, Hallym University, Chuncheon 24252, Republic of Korea; 9 Eye Unit, “Luigi Curto” Hospital, Azienda Sanitaria Locale Salerno, 84035 Polla, Italy; i.depascale@aslsalerno.it (I.D.P.); al.pellegrino@aslsalerno.it (A.P.)

**Keywords:** anthocyanins, curcumin, epigallocatechin gallate, eye diseases, flavonoids, polyphenols, quercetin, resveratrol

## Abstract

The worldwide burden of visual impairment is mostly attributed to ocular diseases, which include alterations of the retina, optic nerve, cornea, and ocular surface. Despite advances in medicine, the prevention and management of these conditions remain challenging, driving ongoing research into novel bioactive compounds. Among these, polyphenols—an assorted group of plant-derived compounds abundant in the human diet—have attracted significant attention due to their potential antioxidant, anti-inflammatory, and immunomodulatory properties. Increasing evidence suggests that polyphenols may help counteract oxidative stress and modulate pathways involved in ocular pathologies, assisting the conventional therapeutic strategies. This narrative review aims to analyze the current evidence about the potential therapeutic effects of the main polyphenols (e.g., curcumin, resveratrol, epigallocatechin gallate) currently recognized for the management of ocular diseases.

## 1. Introduction

Nowadays, one of the goals of the United Nations Summit on Sustainable Development is to improve eye health, which may be accomplished by including measures for care, prevention, promotion, and rehabilitation [1]. In fact, enhancing eye health involves more than just having the finest eyesight; it also entails lowering impairment and raising wellbeing [2]. Age, local and specific diseases, excessive use of digital devices or concurrent pathologies are all causes of eye disorders. In addition to having a detrimental impact on everyday activities and quality of life, impaired vision also raises the likelihood of depression and disability in later life and can result in a loss of independence [3,4].

**Figure 1:**
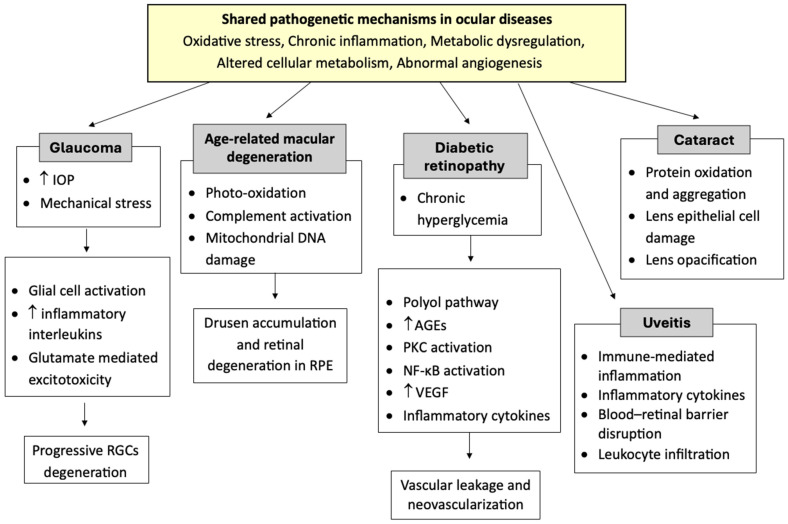
Schematization of the shared pathogenetic mechanism in ocular diseases. ↑: increase; IOP: intraocular pressure; RGCs: retinal ganglion cells; RPE: retinal pigment epithelium; AGEs: advanced glycation end-products; PKC: protein kinase C; NF-κB: nuclear factor kappa-light-chain-enhancer of activated B cells; VEGF: vascular endothelial growth factor.

Glaucoma, diabetic retinopathy (DR), cataracts, age-related macular degeneration (AMD), retinal detachment, uveitis, uncorrected refractive error, and posterior capsular opacification are among the common eye diseases that can negatively affect the quality of life [5,6]. Their multifactorial etiologies often involve a complex interplay of oxidative stress, inflammatory cytokines, dysregulated angiogenesis, and altered cellular metabolism (Figure 1).

For example, elevated intraocular pressure (IOP) and mechanical stress in glaucoma lead to activation of glial cells, release of interleukins, and glutamate excitotoxicity pathways affecting retinal ganglion cells [7,8]. In DR, chronic hyperglycemia activates the polyol pathway, advanced glycation end-products, protein kinase C, and Nuclear Factor kappa-light-chain-enhancer of activated B cells (NF-κB) signaling, contributing to vascular leakage and neovascularization via upregulation of vascular endothelial growth factor (VEGF) and inflammatory mediators [9]. In AMD, chronic photo-oxidation, complement system activation, and mitochondrial DNA damage in the retinal pigment epithelium (RPE) promote drusen accumulation and retinal degeneration [10,11]. Increasing evidence suggests that diet and specific nutrients may modulate these interconnected molecular networks, complementing standard pharmacological or surgical treatments and potentially slowing disease progression [12,13,14].

Several micronutrients and nutraceutical compounds—such as vitamins, carotenoids, omega-3 fatty acids, trace elements, and plant-derived molecules—can influence cellular homeostasis in the eye by regulating oxidative balance, inflammatory cascades, metabolic signaling, and photoreceptor health [15,16,17]. To support their usage in clinical practice, it is crucial to investigate the primary natural antioxidant molecules, as oxidative stress is a common denominator in the development and progression of many ocular diseases [18]. For example, key endogenous antioxidant pathways such as the Kelch-like ECH-associated protein 1/Nuclear factor erythroid 2-related factor 2 (Keap1/Nrf2) signaling system, responsible for the transcription of protective enzymes like heme oxygenase-1 (HO-1), glutathione peroxidase, and superoxide dismutase (SOD), are often impaired in ocular pathology [19]. Likewise, chronic activation of inflammatory transcription factors such as NF-κB contributes to cytokine release, microglial activation, and angiogenic drift [20,21]. For this reason, modulating these pathways through nutritional or nutraceutical interventions has emerged as a highly promising therapeutic approach.

Among these bioactive antioxidant molecules, polyphenols are a heterogeneous class of plant-derived secondary metabolites that are characterized by multiple hydroxylated aromatic rings. They represent one of the most abundant groups of bioactive compounds in the human diet, being widely distributed in fruits, vegetables, tea, coffee, wine, cocoa, and numerous medicinal plants [22,23,24]. Polyphenols are commonly divided into two primary categories: flavonoids and non-flavonoids. To date, over 8000 distinct phenolic compounds have been identified, of which more than 4000 belong to the flavonoid family [25]. Non-flavonoid polyphenols include phenolic acids (e.g., ferulic acid), stilbenes (e.g., resveratrol), curcuminoids (e.g., curcumin), lignans, and other related compounds. Flavonoids are further subdivided into six principal subgroups: flavones, flavonols, flavanols, flavanones, isoflavones, and anthocyanins [25]. They have antioxidant properties thanks to their numerous phenolic hydroxyl groups. In addition, they possess other beneficial properties, including antibacterial and anti-inflammatory effects [26,27]. In the eye, polyphenols can act on several key structures, including the cornea, lens, retina, choroid, optic nerve, and RPE. They may preserve photoreceptor stability, enhance mitochondrial bioenergetics, reduce microglial activation, prevent pericyte loss in DR, and maintain barrier integrity of the blood–retina barrier [28,29,30,31]. Experimental evidence further suggests that polyphenols can attenuate RPE senescence, inhibit amyloid-like protein aggregation present in AMD drusen, promote autophagic clearance, and reduce ferroptotic damage by modulating iron-related oxidative pathways. These multifaceted activities allow polyphenols to support tissue repair, maintain physiological retinal function, and counteract degenerative processes under pathological conditions [28,29,30,31].

Given the growing scientific interest and the increasing number of polyphenolic compounds being investigated in ophthalmology, an up-to-date and comprehensive synthesis is timely (Table 1). For this reason, the purpose of the present narrative review is to consolidate the current knowledge on the major polyphenols that have demonstrated potential therapeutic effects in ocular diseases, with a proven ophthalmological relevance, and that are currently used for the management of eye disorders, summarizing experimental findings, mechanistic insights, and available clinical evidence. This overview may help guide future research and support the rationale for integrating polyphenol-based strategies into preventive or adjunctive ophthalmic care.

## 2. Materials and Methods

A comprehensive search of the discussed topic was carried out on the Scopus and PubMed medical databases. The earliest publication date was set at January 1980, and the search ended in August 2025. The database search strategy was formulated around the term “Eye diseases” (such as “glaucoma”, “age-related macular degeneration”, “dry eye diseases”, “diabetic retinopathy”, etc.) and several other terms regarding the main polyphenols (“Anthocyanins”, “Baicalin”, “Curcumin”, “Epigallocatechin gallate”, “Quercetin”, “Resveratrol”, “Ferulic acid”). The existing literature and/or information gleaned from linked bibliographies were considered while choosing the search keywords. Only full articles and case reports were reviewed, while unrelated and duplicate papers were not included. For extra inclusions, bibliographies from the original searches were also manually added. At last, 119 articles were included in this review. Given the exploratory and narrative nature of the review, no strict methodological framework was applied, though transparency in search strategy was maintained.

## 3. Anthocyanins

Anthocyanins are water-soluble pigments belonging to the flavonoid family. They are abundant in a variety of fruits and vegetables, including blueberries, blackcurrants, bilberries, and grapes [64]. These polyphenolic compounds have antioxidant, anti-inflammatory, and neuroprotective properties [65] and have been studied in various conditions, including cardiovascular, metabolic, and neurodegenerative diseases [66,67]. There has been increasing interest in their potential role in ocular health, particularly in conditions characterized by oxidative stress and inflammation, such as glaucoma and DR, and in supporting visual function [68]. Anthocyanins exert their antioxidant activity by scavenging reactive oxygen species (ROS), donating hydrogen ions, and modulating signaling pathways such as Nrf2, NF-κB, and Mitogen-Activated Protein Kinases (MAPKs), which regulate oxidative stress and inflammation [69]. Despite their biological potential, the oral bioavailability of anthocyanins remains low. They are unstable at neutral or alkaline pH and sensitive to light, temperature, and oxygen, which promotes degradation and limits their absorption in the gastrointestinal tract [70,71]. They are also extensively metabolized by the liver and gut microbiota, which further limits their systemic effects [72]. Anthocyanins are generally considered safe and well tolerated. Clinical studies administering up to 640 mg/day have reported no serious adverse events, although mild gastrointestinal symptoms have occasionally been observed [73].

These flavonoids can act as neuroprotective agents in glaucomatous optic neuropathy, supporting retinal ganglion cell survival, enhancing ocular perfusion, and contributing to the preservation of visual function [74]. In patients with open-angle glaucoma, daily supplementation with 50 mg of blackcurrant anthocyanins for two years resulted in reduced IOP and slower progression of visual field damage [32]. A shorter intervention with the same dosage over 4 weeks was also demonstrated to increase blood flow to the optic nerve head and peripapillary retina, improving ocular perfusion [33]. In a formulation combining bilberry extract and Ginkgo biloba, anthocyanins contributed to improved visual function in patients with normal tension glaucoma [75]. In mice, oral administration of bilberry extract (100–500 mg/kg/day) suppressed retinal ganglion cell (RGC) death after optic nerve crush, suggesting a potential role in retinal damage [76].

Blueberry and bilberry anthocyanins also appear to play a role in diabetes and its complications, particularly DR [34,77]. While some clinical studies suggested that high doses of blueberry anthocyanins may support glycemic control and contribute to the prevention or management of type 2 diabetes [78], the majority of available evidence focused on their protective effects against microvascular diabetic complications. In fact, anthocyanins may help to modulate retinal inflammation and oxidative stress, key mechanisms in DR pathogenesis [77]. In a clinical study of 30 patients with intermediate AMD, a 6-month supplementation with Macuprev^®^ (a formulation containing 90 mg of anthocyanosides) improved macular pre-ganglionic function, potentially reducing inflammation associated with DR lesions [79]. In experimental models, blueberry anthocyanin extracts protected diabetic rat retinas from high-glucose–induced oxidative stress and inflammation by upregulating the Nrf2/Heme oxygenase-1 (HO-1) antioxidant pathway [80]. Furthermore, bilberry extract has demonstrated the ability to protect the blood-retinal barrier in diabetic rats by downregulating VEGF expression and preserving tight junction proteins, thus potentially preventing macular edema and vision loss in advanced stages of DR [35].

Anthocyanin supplementation has also shown potential in supporting various aspects of visual function, particularly in cases of visual fatigue or accommodative stress. In a randomized controlled trial, a 6-week supplementation containing anthocyanins (36 mg), astaxanthin, and lutein improved accommodative function and reduced eye fatigue symptoms in individuals exposed to visual display terminals [36]. In another study, oral intake of black currant anthocyanosides improved dark adaptation and alleviated symptoms of visual fatigue (asthenopia) [81]. However, no improvements in night visual acuity or contrast sensitivity were observed in a trial using 160 mg/day of bilberry extract for 3 weeks [82], suggesting that further studies are needed to clarify its efficacy in this context.

**Figure 2:**
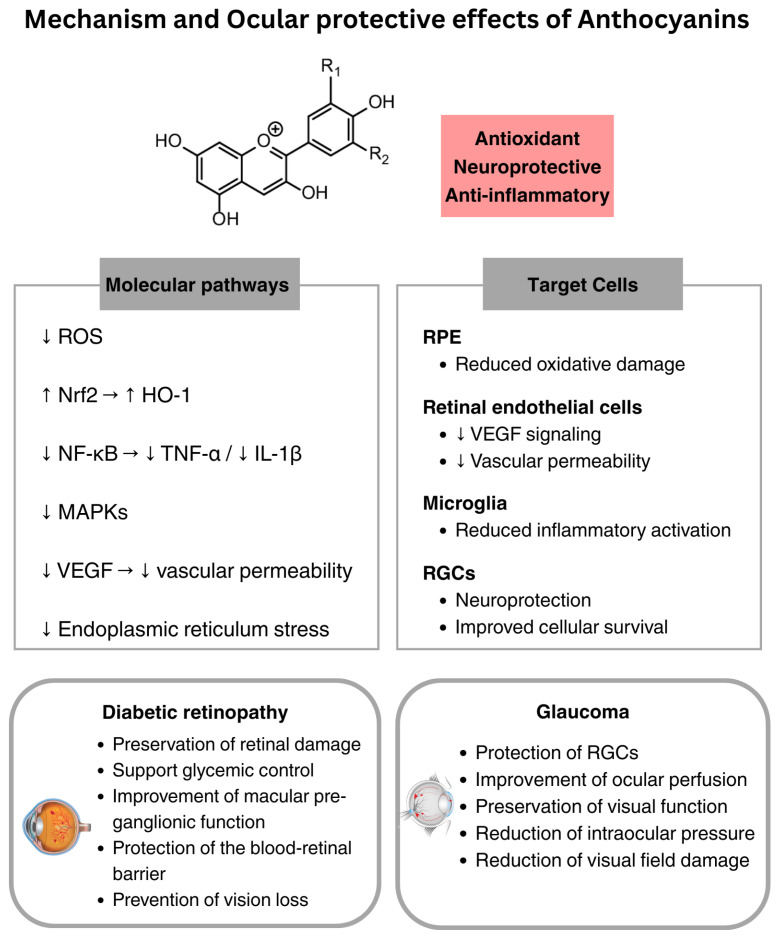
Ocular protective effects of anthocyanins. ↓: decrease; ↑: increase; ROS: reactive oxygen species; Nrf2: nuclear factor erythroid 2-related factor 2; HO-1: heme oxygenase-1; NF-κB: nuclear factor kappa-light-chain-enhancer of activated B cells; TNF-α: tumor necrosis factor α; IL: interleukin; MAPKs: mitogen-activated protein kinases; VEGF: vascular endothelial growth factor; RPE: retinal pigment epithelium; RGCs: retinal ganglion cells.

In conclusion, clinical evidence for anthocyanins supports modest functional benefits, primarily through vascular and neuroprotective mechanisms (Figure 2) (Table 1), but further large-scale trials are needed to have a better understanding of effective dosages and to improve their bioavailability.

## 4. Baicalin

Baicalin is one of the main flavones extracted from the root of *Scutellaria baicalensis* Georgi, a popular medicinal herb in China and other Asian countries. The dried root of this plant is used to treat flu, diarrhea, headaches, and abdominal pain [83]. Baicalin exhibits antioxidant, anti-inflammatory, neuroprotective, and immunomodulatory properties by inhibiting the activation of nuclear factor NF-κB and the Nucleotide-Binding Domain, Leucine-Rich Repeat and Pyrin Domain-Containing protein 3 (NLRP3) inflammasome, as well as by suppressing the expression of pro-inflammatory factors, such as interleukin (IL) 1β, IL-6, IL-8, Tumor Necrosis Factor α (TNF-α), and cyclooxygenase 2 [84,85,86]. Baicalin demonstrates promising properties in preclinical eye disease models—including uveitis, glaucoma, and retinal neovascularization [87]. However, translation into clinical ophthalmology will require further studies to address its bioavailability, safety profile, and efficacy in human trials [86].

A recent study found that baicalin can significantly reduce inflammation in experimental autoimmune uveitis by modulating the Hypoxia-Inducible Factor 1α (HIF-1α) pathway and balancing macrophage polarization between pro-inflammatory M1 and anti-inflammatory M2, while improving mitochondrial function and lowering oxidative stress [37]. In addition, a study by Jung et al. [38] evaluated the neuroprotective effects of baicalin against retinal ischemia/reperfusion injury in rats and against various insults in RGC-5 cells in vitro. Intraperitoneal administration of baicalin before and after ischemic insult significantly mitigated alterations in retinal protein and mRNA expression, preserved antigen localization, and reduced oxidative stress. In vitro, baicalin attenuated cell death induced by light exposure, hydrogen peroxide, and serum deprivation, with its cytoprotective properties. In an experimental animal model, baicalin intravitreal injection reduced laser-induced choroidal neovascularization and vascular leakage in a dose-dependent manner, attenuating the up-regulation of VEGF, platelet-derived growth factor (PDGF), and matrix metalloproteinase-2 (MMP-2), suggesting its potential for treating exudative AMD [39]. In conclusion, this bioactive compound has shown very promising preclinical results for its anti-inflammatory and neuroprotective effects (Table 1), but human clinical trials are needed to better understand its dosages, bioavailability, and safety.

## 5. Curcumin

Curcumin is a polyphenolic compound derived from the rhizomes of *Curcuma longa*, and it is the principal curcuminoid responsible for the characteristic yellow color of turmeric. Historically utilized in Chinese traditional medicine, curcumin has garnered attention for its pleiotropic pharmacological properties, including anti-inflammatory, antioxidant, antimicrobial, anti-angiogenic, neuroprotective effects, and antitumor activities [88,89,90,91]. Given these properties, curcumin has been investigated as a potential therapeutic agent for various diseases, including diabetes, neurodegenerative diseases, and cancer [91,92,93].

In ophthalmology, research has primarily focused on its potential benefits in retinal diseases, such as DR, AMD, and retinal neurodegeneration, where oxidative stress and chronic inflammation play a pivotal role in disease progression [12,13,94,95]. Curcumin exerts its biological effects by modulating several molecular pathways: it can downregulate the nuclear factor NF-κB, which leads to decreased production of pro-inflammatory cytokines (e.g., TNF-α, IL-1β, IL-6); it can activate the Nrf2/HO-1 pathway, which enhances cellular antioxidant defenses and helps mitigate oxidative stress [88], it can reduce the expression of oxidative stress biomarkers, including SOD and glutathione [96], and it can downregulate the VEGF expression by inhibiting neovascularization [95]. Numerous clinical studies have demonstrated curcumin’s favorable safety profile, showing its good tolerability and efficacy, even at relatively high oral doses ranging from 4 to 8 g per day [44]. However, its clinical application is limited by significant pharmacokinetic challenges, such as poor water solubility, light sensitivity, low bioavailability, limited absorption, rapid systemic metabolism and elimination, and its narrow effective dose range [97,98,99,100]. For all these reasons, curcumin should be well-examined in the future for further studies on its efficacy in the case of ocular diseases.

Curcumin has been shown to potentially protect the retina in vivo, preserving retinal function, reducing oxidative stress, inflammation, and apoptosis, and slowing degeneration in AMD and light-induced retinal damage [40]. In dry AMD patients, oral curcumin (1330 mg twice daily for 6 months) was demonstrated to reduce drusen volume in most subjects, decrease mean foveal volume, and, in some cases, improve disease stage [41]. Evidence from a recent retrospective cohort study indicates that curcuma-based nutritional supplements (CBNS) may confer a protective effect against both the onset and progression of AMD [101]. The study analyzed data from over 1.8 million patients, approximately 66,800 of whom were CBNS users. Statistical comparisons revealed that CBNS users had significantly lower risks of developing nonexudative/advanced AMD or geographic atrophy, blindness, and the need for intravitreal anti-VEGF therapy compared with non-users. Among patients with early-stage nonexudative AMD, CBNS use was also associated with reduced progression to advanced disease. These results support the potential of curcumin as a protective agent in AMD, likely due to its anti-inflammatory and antioxidant properties. Another recent clinical investigation evaluated the adjunctive use of a CBNS in combination with anti-VEGF therapy for neovascular AMD [102]. Patients receiving both treatments demonstrated superior visual outcomes and required fewer intravitreal injections compared with those on anti-VEGF monotherapy. The supplementation was well tolerated, supporting curcumin’s potential role as a safe and effective adjuvant that may enhance functional results and extend treatment intervals.

A range of in vitro and in vivo studies has demonstrated the beneficial effects of curcumin at different stages of DR [103]. In streptozotocin-induced diabetic rat models, curcumin (0.5–1 g/kg for 6–16 weeks) prevented oxidative DNA damage, reduced nitrotyrosine levels, restored glutathione, inhibited inflammatory mediators (IL-1β, TNF-α, NF-κB), and lowered VEGF expression [42,104]. It also improved antioxidant enzyme activity (SOD, catalase) and prevented retinal vascular dilation and tortuosity [42,104]. Moreover, lower doses (200 mg/kg) seem to promote regeneration of damaged choroidal microvasculature, repairing shrinkage, constriction, microaneurysms, and vessel blind endings [105].

A randomized, double-blind, placebo-controlled clinical trial investigated the effects of curcumin in combination with piperine (1010 mg/day) over 12 weeks in patients with non-proliferative DR [106]. Though there were no significant changes in optical coherence tomography or optical coherence tomography angiography metrics, the treatment group showed a significant enhancement in antioxidant markers and a decrease in oxidative stress [106].

Another recent randomized, double-blind, placebo-controlled clinical trial assessed a proprietary oral supplement blend containing lutein, zeaxanthin, curcumin (200 mg curcuminoids), and vitamin D3 versus placebo over 8 weeks in adults with dry eye disease [43]. The supplement significantly improved tear production, quality, and stability, reduced inflammation and ocular surface damage, and alleviated participants’ symptoms. On the other hand, a clinical study performed by Kapil and colleagues evaluated oral bio-enhanced curcumin added to standard topical therapy in mild-to-moderate dry eye disease patients. Over 3 months, they found that curcumin supplementation effectively improved the tear film stability, lipid layer thickness, and reduced the bulbar redness [107].

Curcumin was also demonstrated to protect retinal neurons and microvessels in retinal ischemia–reperfusion injury by inhibiting the NF-κB and signal transducer and activator of transcription 3 (STAT3) pathways [108]. In proliferative vitreoretinopathy, curcumin may inhibit RPE proliferation and induce apoptosis and necrosis [109]. Curcumin also appears to possess anti-cancer activity in retinoblastoma cell lines by promoting apoptosis and blocking proliferation [110,111].

**Figure 3:**
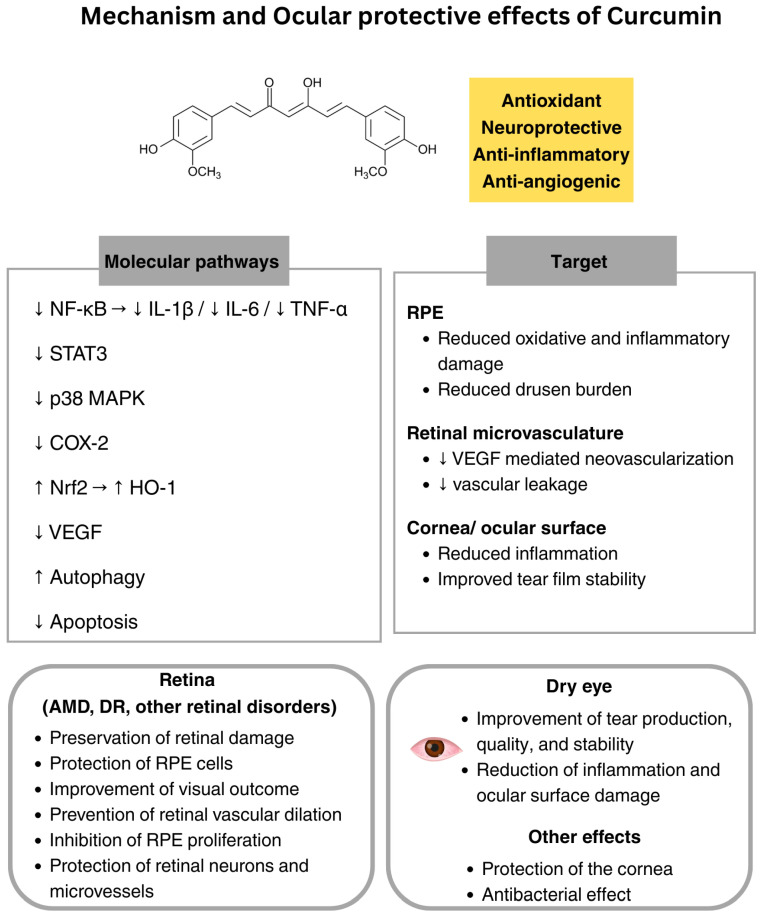
Ocular protective effects of curcumin. ↓: decrease; ↑: increase; NF-κB: nuclear factor kappa-light-chain-enhancer of activated B cells; IL: interleukin; TNF-α: tumor necrosis factor α; STAT3: signal transducer and activator of transcription 3; MAPKs: mitogen-activated protein kinases; COX-2: cyclooxygenase-2; Nrf2: nuclear factor erythroid 2-related factor 2; HO-1: heme oxygenase-1; VEGF: vascular endothelial growth factor; RPE: retinal pigment epithelium; AMD: age-related macular degeneration; DR: diabetic retinopathy.

Curcumin could also protect the cornea by modulating oxidative stress, inflammation, and angiogenesis [44]. In fact, curcumin is able to inhibit NF-κB and p38 MAPK, reducing pro-inflammatory cytokines (IL-1β, IL-6, TNF-α) [112], while activating the Keap1/Nrf2 pathway to enhance antioxidant defenses [113]. Furthermore, curcumin also promotes wound healing, reduces fibrosis, and prevents neovascularization to preserve corneal transparency [114,115]. Finally, in bacterial ocular infections, curcumin can disrupt biofilm integrity and efflux pumps, thereby increasing antibiotic susceptibility [45]. In addition, curcumin protects against oxidative stress through Keap1/Nrf2 activation [116] and exhibits anti-inflammatory and antibacterial efficacy [115]. In summary, among all the polyphenols, curcumin is certainly the one with the strongest scientific evidence in human clinical trials, with a potential efficacy for the AMD treatment (Figure 3) (Table 1). However, its low bioavailability represents a significant limitation to its clinical use, and a standardized recommended dose has not yet been achieved.

## 6. Epigallocatechin-3-Gallate

Epigallocatechin-3-gallate (EGCG) is the major catechin in green tea, accounting for approximately 50% of its total polyphenol content [117]. EGCG exerts potent antioxidant, anti-inflammatory, and vasodilatory effects by scavenging free radicals and modulating signaling pathways, including NF-κB, MAPKs, and VEGF [117,118]. These mechanisms support its neuroprotective potential, attracting interest for applications in ocular diseases, including glaucoma and other retinal diseases [14,119]. However, the availability of clinical studies, as well as in vivo and in vitro research on EGCG in these contexts, is still limited. EGCG has poor oral bioavailability, primarily due to rapid degradation and metabolism in the gastrointestinal tract [117,118]. Although generally well tolerated, high doses have occasionally been linked to mild gastrointestinal symptoms or rare hepatotoxicity [120].

In a short-term clinical trial, EGCG supplementation improved inner retinal function in patients with open-angle glaucoma, with no effect seen in ocular hypertension or visual field tests [121]. A randomized clinical trial in 43 healthy volunteers showed that oral intake of green tea extract or EGCG (400 mg) significantly lowered IOP within 90 min [122]. In an optic nerve crush mouse model, EGCG was demonstrated to protect RGCs from survival, probably by suppressing oxidative damage and apoptosis [46].

EGCG was also proposed as an adjunct to anti-VEGFA therapy for wet AMD, based on its ability to down-regulate VEGFA expression, inhibit MAPK1/3 signaling, and protect retinal cells from oxidative damage [47]. These therapeutic properties suggest that EGCG may enhance anti-VEGF efficacy and mitigate potential side effects. Nevertheless, clinical trials are necessary to determine if EGCG may benefit patients undergoing anti-VEGF therapy.

In a study on human retinal endothelial cells exposed to high glucose, EGCG reduced apoptosis and inflammatory cytokine levels, such as TNF-α, IL-6, and intercellular adhesion molecule 1 (ICAM-1), while downregulating the MAPK/Extracellular signal-Regulated Kinase (ERK) and VEGF pathways [107]. To summarize, EGCG showed promising preclinical and early clinical data, with a need for long-term trials to better understand its potential use in a clinical setting (Table 1).

## 7. Ferulic Acid

Ferulic acid is a plant-derived phenolic compound with antioxidant and anti-inflammatory properties [123]. It can protect cells by reducing ROS production, inhibiting aldose reductase, and activating Phosphatidylinositol 3-kinase (PI3K)/Protein kinase B (Akt) and Nrf2/HO-1 pathways, which enhance antioxidant defenses [123]. Its anti-inflammatory effects are also linked to Peroxisome Proliferator-Activated Receptor gamma (PPARγ) activation and downregulation of NF-κB, ICAMs, and p38 MAPK signaling [123]. All these properties make it a promising nutraceutical for the prevention of chronic diseases, including ocular disorders. However, its clinical application is limited by poor oral bioavailability, and further human studies are needed to clarify pharmacokinetics, efficacy, and safety.

In human retinal pigment epithelial cell models exposed to hydrogen peroxide, pre-treatment with ferulic acid promoted cell survival, reduced apoptosis, and restored antioxidant enzyme activity. This finding points out the ferulic acid potential to safeguard RPE cells from oxidative stress implicated in AMD [61]. In diabetic mouse models, oral administration of ferulic acid attenuated high glucose-induced apoptosis in retinal pigment epithelium cells, contributing to structural and functional protection of retinal layers [62]. In human lens epithelial cells, ferulic acid protected against ultraviolet A-induced oxidative stress and apoptosis by activating the Keap1/Nrf2 signaling pathway, thereby reducing lens opacification and suggesting a potential role in preventing and mitigating cataracts induced by ultraviolet A radiation [63].

In conclusion, ferulic acid is a promising polyphenol for the treatment of ocular diseases, with potential antioxidant effects demonstrated in vitro and in vivo (Table 1). However, no clinical trials have yet been carried out on humans, and there is insufficient data regarding its bioavailability, efficacy, and safety.

## 8. Quercetin

Quercetin is one of the most abundant flavonols found in various fruits and vegetables, particularly in onions, apples, berries, and kale. It is known for its antioxidant, anti-inflammatory, and neuroprotective properties by scavenging ROS and modulating inflammatory and apoptotic pathways such as NF-κB and MAPKs [124]. Emerging evidence has highlighted the potential of quercetin for promoting ocular health. In fact, it may counteract the mechanisms involved in the pathogenesis of AMD, glaucoma, DR, and cataract formation [27]. At a molecular level, quercetin seems to majorly interact with sirtuins, especially sirtuin 1 and sirtuin 6, thus modulating numerous signaling pathways, contributing to its therapeutic effects. These pathways play crucial roles in reducing ROS, inflammation, autophagy regulation, mitochondrial biogenesis, glucose utilization, fatty acid oxidation, and genome stability [125]. However, most of the evidence only comes from in vitro and animal models, and clinical studies are still limited. Moreover, its oral bioavailability remains low due to poor water solubility and rapid metabolism.

In rat models of chronic glaucoma, quercetin has been shown to directly protect retinal ganglion cells by enhancing inhibitory neurotransmission and decreasing excitatory input, thereby reducing excitotoxic damage and supporting cell survival [50]. Additionally, in a rat model of chronic ocular hypertension, oral quercetin administration preserved RGC survival and function, probably by enhancing mitochondrial performance and inhibiting apoptosis, independently of IOP reduction [51].

In DR, quercetin may help preserve retinal structure and function by reducing oxidative stress, inflammation, and neovascularization [126]. In a study on diabetic rats, quercetin (25 and 50 mg/kg for six months) reduced retinal oxidative stress, inflammation, and cell death, improving retinal thickness and increasing survival of RGCs [127]. In another study on diabetic rats, quercetin treatment also promoted neuroprotection by increasing neurotrophic factors and preserving retinal integrity [49]. In vitro and in vivo studies have also shown that quercetin may inhibit choroidal neovascularization and enhance choroidal blood flow, supporting its promising role in managing both dry and wet forms of AMD [52].

Finally, a recent study evaluated an ophthalmic formulation based on olive leaf extract and identified the quercetin derivative dihydroquercetin as a key active compound involved in promoting corneal epithelial wound healing. The formulation exhibited antioxidant and anti-inflammatory properties, resulting in accelerated corneal re-epithelialization in vivo [128]. In summary, like other polyphenols, quercetin demonstrated a strong potential mechanistic rationale and robust preclinical support (Table 1), but to date, clinical studies are lacking.

## 9. Resveratrol

Resveratrol is a non-flavonoid polyphenol belonging to the stilbenes class. It is a natural compound produced by plants against pathogens and environmental stressors. The main dietary sources of resveratrol are grape skins, berries, and peanuts, and it is a component of red wine. Interest in resveratrol grew among scientists in the early 1980s when epidemiological studies revealed that the French population had a lower mortality rate from coronary heart disease, despite lifestyle factors associated with a higher risk, such as a diet high in saturated fats [129]. This phenomenon, known as the “French paradox”, was partially attributed to moderate consumption of red wine, which contains resveratrol as a bioactive compound [130]. Resveratrol is now recognized for its pleiotropic properties, including antioxidant, anti-inflammatory, anti-apoptotic, and anti-aging effects [131]. These properties have been studied in various pathological conditions, from cardiovascular diseases to cancer and neurodegenerative disorders [132,133,134]. Resveratrol exerts its beneficial effects by modulating several cellular pathways and molecular targets, such as reducing ROS, inhibiting pro-inflammatory cytokines (e.g., TNF-α, IL-6), regulating lipid peroxidation, promoting autophagy, and suppressing apoptosis [135]. Due to its mechanisms of action, resveratrol has gained attention as a promising candidate for the prevention and treatment of ocular diseases, particularly age-related ocular diseases, glaucoma, cataract, and DR [136,137,138]. Like quercetin, resveratrol also appears to perform its therapeutic effects by interacting with the sirtuins’ pathways, thus promoting a reduction in oxidative stress [139]. However, resveratrol’s low bioavailability (<1%) is a major limitation of its clinical application. After oral administration, resveratrol is rapidly metabolized in the liver and intestine to sulfate and glucuronide conjugates, which significantly reduce its systemic bioactivity [140]. Resveratrol is generally considered safe and well-tolerated at moderate doses [141], though high doses (2.5–5 g/day) have been associated with mild to moderate gastrointestinal symptoms over prolonged administration [142].

In vitro, resveratrol has been shown to protect RPE cells from oxidative stress induced by hydrogen peroxide and from hyperproliferation by inhibiting mitogen-activated protein kinase (MAPK) signaling cascade, and ERK 1/2 activities [53]. Additionally, resveratrol reduced intracellular ROS levels by enhancing the activities of SOD, glutathione peroxidase, and catalase in human RPE cells [143]. It is hypothesized that resveratrol may help control pathological choroidal neovascularization in AMD by suppressing VEGF secretion induced by inflammatory cytokines and hypoxia [144]. However, further studies in animal models and clinical trials are needed to confirm these effects.

Resveratrol supplementation in diabetic animal models has been shown to improve hyperglycemia and retinal complications by reducing oxidative stress markers, increasing SOD activity, and mitigating both NF-κB activation and retinal cell apoptosis [54]. Another animal study found that resveratrol treatment decreased vascular lesions and VEGF induction in the retinas of diabetic mice [145]. Furthermore, it seems that resveratrol may protect against retinal vascular degeneration by reducing endoplasmic reticulum stress [146]. A 6-month clinical trial involving 99 patients assessed the antioxidant effectiveness of a nutraceutical formulation containing grape pomace extract, rich in resveratrol, for treating DR. The results showed that the supplement effectively reduced retinal swelling and oxidative stress, improving visual outcomes in DR patients [147].

Intravitreal administration of resveratrol in a microbead-induced high IOP mouse model was shown by Cao et al. [56] to protect RGCs by reducing ROS generation and to delay the progression of visual dysfunction. In two other studies on animal models, resveratrol also reduced the loss of RGCs and improved retinal function in mice after ischemia–reperfusion injury, likely by inhibiting pro-apoptotic and angiogenic pathways [148,149].

**Figure 4:**
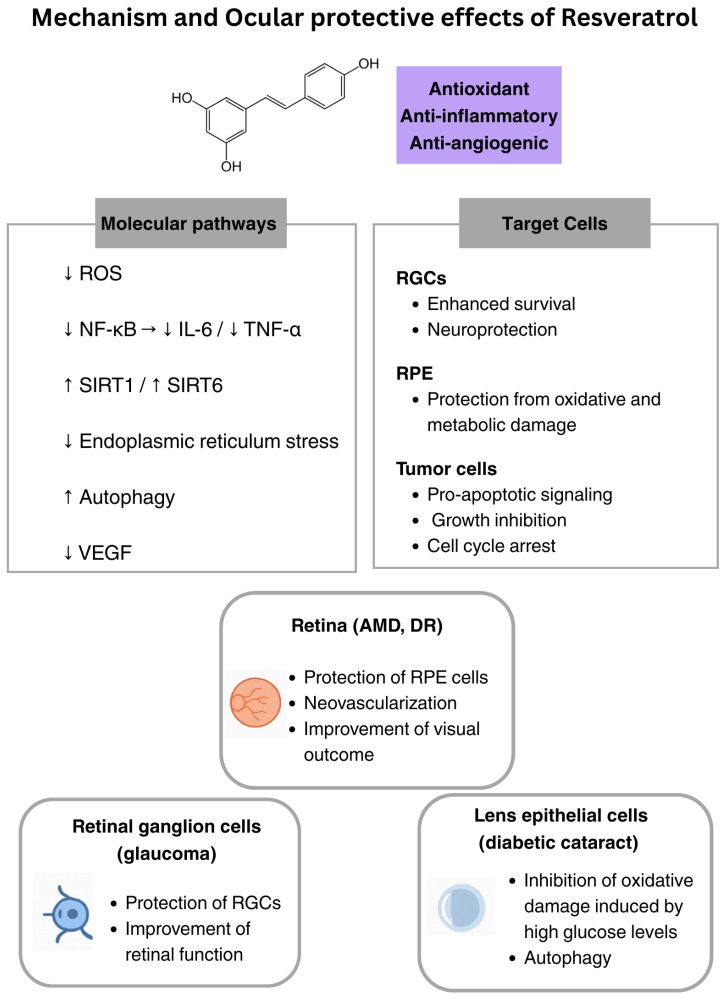
Ocular protective effects of resveratrol. ↓: decrease; ↑: increase; ROS: reactive oxygen species; NF-κB: nuclear factor kappa-light-chain-enhancer of activated B cells; IL: interleukin; TNF-α: tumor necrosis factor α; SIRT: sirtuin; VEGF: vascular endothelial growth factor; RGCs: retinal ganglion cells; RPE: retinal pigment epithelium; AMD: age-related macular degeneration; DR: diabetic retinopathy.

Some studies have also suggested that resveratrol may play a preventive role in other eye-related diseases. In human lens epithelial cells, resveratrol inhibited oxidative damage induced by high glucose levels by promoting autophagy, providing a theoretical basis for its use in the prevention and treatment of diabetic cataracts [55]. In uveitis models, resveratrol reduced inflammation by inhibiting oxidative damage and NF-κB activation [57]. Resveratrol was also demonstrated to induce tumor cell death and inhibit the growth of eye cancers, including uveal melanoma, by triggering mitochondrial dysfunction and caspase-3 activation [58]. In addition, it can also cause cell cycle arrest and apoptosis in a time- and dose-dependent manner in retinoblastoma [150]. Finally, due to its anti-inflammatory properties, resveratrol has demonstrated beneficial effects on both myopia [59] and dry eye disease [60], suggesting its potential as a candidate for treating these conditions. To summarize, after curcumin, resveratrol is one of the most versatile polyphenols, with several clinical data available in the case of ocular diseases (Figure 4) (Table 1), but its clinical use is strongly limited by its very low bioavailability.

## 10. Discussion

This narrative review highlights the growing scientific interest in polyphenols as multifunctional bioactive compounds capable of modulating key pathological pathways implicated in major ocular diseases. Across the extensive body of preclinical literature, a unifying theme emerges; polyphenols consistently exert antioxidant, anti-inflammatory, anti-apoptotic, and, in several cases, anti-angiogenic effects. These mechanisms converge on molecular targets such as NF-κB, Nrf2/HO-1, MAPKs, STAT3, VEGF, and sirtuin-mediated pathways, suggesting that the therapeutic potential of polyphenols extends beyond simple free-radical scavenging and encompasses broader regulation of cellular homeostasis. The evidence summarized in this review indicates that anthocyanins, baicalin, curcumin, EGCG, quercetin, ferulic acid, and resveratrol act on overlapping yet complementary biological processes, creating a compelling rationale for their consideration as adjuvant agents in retinal, optic nerve, ocular surface, and lens disorders.

A strength of this review lies in its comprehensive overview of both the biological underpinnings and the disease-specific effects of individual polyphenols. By integrating findings from mechanistic studies, animal models, and the available clinical literature, the review provides a unified and up-to-date perspective on how various classes of polyphenols may contribute to neuroprotection in glaucoma, vascular and inflammatory modulation in DR and AMD, and tear film and epithelial support in ocular surface diseases. Importantly, the included evidence underscores that some polyphenols—such as curcumin and anthocyanins—have reached a more advanced stage of clinical evaluation, with encouraging preliminary results. These findings strengthen the growing hypothesis that dietary or supplemental polyphenols could serve as potential low-risk, accessible, and biologically meaningful adjuncts to conventional ophthalmic therapies.

Moreover, the review emphasizes an important conceptual strength: the multitarget nature of polyphenols. Given that most ocular diseases—especially AMD, DR, glaucoma, and dry eye disease—are multifactorial and driven by a combination of oxidative stress, chronic inflammation, mitochondrial dysfunction, vascular dysregulation, and neurodegeneration, agents capable of modulating multiple pathways simultaneously represent a particularly promising therapeutic class. This multidimensional profile may also explain why polyphenols appear to enhance or complement established treatments, such as anti-VEGF therapy or topical ocular surface medications, in several early studies. As the field moves forward, integrating polyphenols into multimodal treatment strategies may prove especially advantageous.

## 11. Safety Considerations

Safety profiles of polyphenols in the context of ocular diseases are not yet fully established, as available data are fragmented and often derived from small or short-term clinical studies. Overall, most compounds reviewed here appear to be well tolerated at dietary or commonly supplemented doses. However, several considerations should be emphasized.

Anthocyanins have demonstrated good tolerability in human trials, with doses up to 640 mg/day generally associated with only mild gastrointestinal discomfort [73]. Curcumin is widely regarded as safe, even at relatively high oral intakes, although rare cases of gastrointestinal symptoms and hepatotoxicity have been reported at very high or prolonged doses, particularly when combined with bioavailability enhancers [97,98,99,100]. EGCG is typically well tolerated but has been associated with mild gastrointestinal effects and, in rare cases, hepatotoxicity, especially at high concentrations or in concentrated extract form [120]. Resveratrol also presents a favorable safety profile at moderate doses, while higher doses (>2.5 g/day) may cause gastrointestinal complaints [142]. For quercetin, human safety data remain limited, and most evidence is derived from preclinical studies. Baicalin and ferulic acid have shown promising biological activity but lack adequate clinical safety data, making it difficult to draw definitive conclusions regarding their tolerability in ophthalmic applications.

Importantly, most available studies do not systematically assess adverse events or potential interactions with standard ophthalmic or systemic medications. Variability in polyphenol formulations, purity, and excipients further complicates the interpretation of safety findings. Long-term safety, particularly in older adults and in patients with comorbidities or polypharmacy, remains insufficiently explored. As interest in nutraceutical interventions grows, future studies should incorporate rigorous monitoring of adverse events, standardized safety endpoints, and assessments of potential drug–nutrient interactions. Establishing clear safety profiles will be essential for the responsible clinical integration of polyphenols in ocular health.

## 12. Limitations

This review has several limitations that should be considered when interpreting its findings. Although a substantial body of preclinical evidence supports the biological plausibility of polyphenols in ocular diseases, most data derive from in vitro or animal models that do not fully replicate human physiology, dosing conditions, or metabolic pathways. Additionally, the complexity of polyphenol metabolism—including interaction with gut microbiota, first-pass metabolism, and formation of active or inactive metabolites—remains poorly understood in the context of ocular tissue exposure. These gaps highlight the need for rigorous pharmacokinetic studies specifically addressing ocular biodistribution and metabolite activity.

Another major limitation across all compounds is their inherently low oral bioavailability, which results from poor solubility, rapid metabolism, and limited tissue distribution. Consequently, the effective concentrations observed in experimental settings may not be achievable through conventional oral supplementation. Moreover, clinical studies remain limited in number and heterogeneous in design, in addition to small sample sizes. Variability in formulations, dosages, intervention duration, and outcome measures restricts comparability across trials and prevents the establishment of standardized recommendations (Table 2). In many cases, polyphenols are administered as part of multi-ingredient supplements, making it difficult to isolate the contribution of individual compounds [43,79].

In addition, although polyphenols are widely consumed as part of a balanced diet, several factors limit the extent to which dietary intake alone can achieve concentrations that are biologically meaningful for ocular protection. In fact, the polyphenol content of foods varies markedly according to cultivar, ripeness, edaphoclimatic conditions, agricultural practices, storage, and processing methods, leading to substantial variability in actual intake [151]. Furthermore, extraction techniques used in supplements—such as solvent extraction, fermentation, or purification steps—can result in products with highly different concentrations and bioactive profiles, complicating comparisons across studies and limiting generalizability [152].

Finally, the strictly narrative nature of this review and reliance on only two scientific databases (PubMed and Scopus) may introduce selection bias. Although efforts were made to ensure a comprehensive search, relevant studies may have been missed, and the predominance of positive findings in the published literature raises the possibility of publication bias.

## 13. Future Directions

Future research should focus on well-designed randomized controlled trials with larger sample sizes, standardized formulations, and harmonized clinical endpoints. Establishing optimal dosing strategies and treatment durations will be essential for assessing the true clinical utility of polyphenols. Pharmacokinetic studies specifically addressing ocular penetration, metabolite activity, and interindividual variability—including genetic and microbiota-related factors—are needed to better understand their translational potential.

Innovative delivery approaches, such as nanoformulations, liposomal carriers, micelles, or prodrug systems, may help overcome current bioavailability challenges [153,154,155,156]. Comparative trials evaluating conventional and enhanced formulations could further clarify whether improved delivery leads to meaningful clinical benefits.

Further investigation is also warranted to explore potential synergistic effects between polyphenols and established ophthalmic treatments, particularly in multifactorial diseases such as AMD, DR, glaucoma, and ocular surface disorders. Finally, studies addressing safety, tolerability, and possible interactions with standard therapies will be critical for supporting their integration into evidence-based clinical practice.

## 14. Conclusions

In conclusion, this narrative review consolidates current knowledge on the therapeutic promise of dietary polyphenols in ocular diseases and highlights their substantial biological relevance across multiple pathogenic pathways. While preclinical findings are compelling and early clinical results are encouraging, significant challenges—including poor bioavailability, limited human evidence, and heterogeneity in study designs—must be addressed before polyphenols can be fully integrated into ophthalmic practice. Advances in formulation science, together with rigorous clinical research, will be essential to harness their full potential. As interest in nutraceutical and preventive ophthalmology continues to grow, polyphenols represent a promising and biologically plausible avenue worthy of further, carefully structured investigation.

## Figures and Tables

**Table 1:** Overview of the main polyphenols used for ocular diseases and discussed in the present review.

Polyphenols	Sources	Effects	Mechanisms of Action	Limitations	Ocular Diseases	References
Anthocyanins	Blueberries Blackcurrant Bilberry Grape	Antioxidant Anti-inflammatory Neuroprotection	↓ ROS ↑ ocular perfusion Modulation of Nrf2, NF-κB, MAPKs	Low oral stability Low bioavailability	Glaucoma	[32,33]
					Diabetic retinopathy	[34,35]
					Visual function	[36]
Baicalin	*Scutellaria baicalensis* Georgi	Antioxidant Anti-inflammatory Immunomodulatory Neuroprotection	↓ NF-κB ↓ IL-1β, IL-6, TNF-α ↓ NLRP3	No clinical trials	Uveitis	[37]
					Glaucoma	[38]
					AMD	[39]
Curcumin	Turmeric	Antioxidant Anti-inflammatory Anti-angiogenic Neuroprotective	↓ NF-κB ↓ IL-1β, IL-6, TNF-α ↑ Nrf2/HO-1 ↓ VEGF	Low bioavailability Rapid metabolism	AMD	[40,41]
					Diabetic retinopathy	[42]
					Dry eye disease	[43]
					Corneal diseases	[44]
					Bacterial Ocular Diseases	[45]
Epigallocatechin-3-gallate	Green tea	Antioxidant Anti-inflammatory	↓ NF-κB ↓ MAPKs ↓ VEGF	Low stability Limited clinical data	Glaucoma	[46]
					AMD	[47]
					Diabetic retinopathy	[48]
Quercetin	Onions Apples Berries Kale	Antioxidant Anti-inflammatory Neuroprotection	↓ NF-κB ↓ MAPKs ↓ ROS	Poor solubility and absorption	Diabetic retinopathy	[49]
					Glaucoma	[50,51]
					AMD	[52]
Resveratrol	Red grapes Berries Peanuts	Antioxidant Anti-inflammatory Anti-apoptotic	↓ ROS ↓ NF-κB ↓ IL-6, TNF-α	Bioavailability < 1%	AMD	[53]
					Diabetic retinopathy	[54]
					Diabetic cataract	[55]
					Glaucoma	[56]
					Uveitis	[57]
					Eye Tumors	[58]
					Myopia	[59]
					Dry eye disease	[60]
Ferulic acid	Ranunculaceae and Gramineae	Antioxidant Anti-inflammatory	↑ PI3K/Akt ↑ Nrf2/HO-1 ↓ NF-κB	Poor bioavailability No clinical trials	AMD	[61]
					Diabetic retinopathy	[62]
					Cataract	[63]

Abbreviations: ↓: decrease; ↑: increase; ROS: reactive oxygen species; Nrf2: nuclear factor erythroid 2-related factor 2; NF-κB: nuclear factor kappa-light-chain-enhancer of activated B cells; MAPKs: mitogen-activated protein kinases; IL: interleukin; TNF-α: tumor necrosis factor α; NLRP3: nucleotide-binding domain, leucine-rich repeat and pyrin domain-containing protein 3; HO-1: heme oxygenase-1; VEGF: vascular endothelial growth factor; PI3K: phosphatidylinositol 3-kinase; Akt: protein kinase B; AMD: age-related macular degeneration.

**Table 2:** Clinical studies investigating polyphenols for ocular diseases.

Formulation	Ocular Disease	Subject	Dose	Duration	Effect	References
Blackcurrant anthocyanins	Glaucoma	n = 38 open-angle glaucoma patients (n = 19; n = 19 placebo)	50 mg/day	2 years	↓ IOP slower visual field loss	[32]
Blackcurrant anthocyanins	Glaucoma	n = 12 healthy volunteers n = 21 glaucoma patients (n = 12; n = 9 placebo)	50 mg/day	4 weeks	↓ IOP ↑ optic nerve head blood flow	[33]
Macuprev^®^	Intermediate AMD	n = 30 AMD patients (n = 15; n = 15 placebo)	anthocyanins 90 mg	6 months	↑ macular preganglionic function	[79]
Curcumin	Dry AMD	n = 20 AMD patients (n = 14; n = 6 placebo)	1330 mg × 2/die	6 months	↓ drusen volume, ↓ foveal volume	[41]
Curcumin + piperine	NPDR	n = 60 DR patients (n = 30; n = 30 placebo)	1010 mg/day	12 weeks	↑ antioxidant markers ↓ oxidative stress	[106]
Oral supplement blend (curcumin, lutein, zeaxanthin, vitamin D3)	Dry Eye disease	n = 155 dry eye patients (n = 77; n = 78 placebo)	200 mg curcuminoids/day	8 weeks	↑ tear stability, production, and quality ↓ inflammation	[43]
Curcumin	Dry eye disease	n = 40 dry eye patients (n = 20; n = 20 placebo)	500 mg tablets × 2/die	3 months	↑film stability ↓bulbar redness	[107]
Green tea and ECGC	Glaucoma prevention	n = 43 healthy volunteers (n = 17 green tea; n = 17 ECGC; n = 9 placebo)	400 mg	90 min	↓ IOP	[122]
Grape pomace extract	DR	n = 99 non-proliferative DR patients (n = 49; n = 50 placebo)	400 mg × 2/die	6 months	↓ retinal swelling, ↓ oxidative stress	[147]

Abbreviations: ↓: decrease; ↑: increase; IOP: intraocular pressure; AMD: age-related macular degeneration; NPDR: nonproliferative diabetic retinopathy; DR: diabetic retinopathy; ECGC: epigallocatechin gallate.

## Data Availability

Data sharing is not applicable (only appropriate if no new data is generated or the article describes entirely theoretical research).

## Abbreviations

The following abbreviations are used in this manuscript:

Akt	Protein kinase B
AMD	Age-related macular degeneration
CBNS	Curcuma-based nutritional supplements
DR	Diabetic retinopathy
EGCG	Epigallocatechin-3-gallate
ERK	Extracellular signal-regulated kinase
HIF-1α	Hypoxia-inducible factor 1α
HO-1	Heme oxygenase-1
ICAM-1	Intercellular adhesion molecule 1
IL	Interleukin
IOP	Intraocular pressure
Keap1	Kelch-like ECH-associated protein 1
MAPKs	Mitogen-activated protein kinases
MMP-2	Matrix metalloproteinase-2
NF-κB	Nuclear factor kappa-light-chain-enhancer of activated B cells
NLRP3	Nucleotide-binding domain, leucine-rich repeat, and pyrin domain-containing protein 3
Nrf2	Nuclear factor erythroid 2-related factor 2
PDGF	Platelet-derived growth factor
PI3K	Phosphatidylinositol 3-kinase
PPARγ	Peroxisome proliferator-activated receptor gamma
RGC	Retinal ganglion cell
ROS	Reactive oxygen species
RPE	Retinal pigment epithelium
SOD	Superoxide dismutase
STAT3	Signal transducer and activator of transcription 3
TNF-α	Tumor necrosis factor α
VEGF	Vascular endothelial growth factor
